# Role of Intra- and Peritumoral Budding in the Interdisciplinary Management of Rectal Cancer Patients

**DOI:** 10.1155/2012/795945

**Published:** 2012-07-31

**Authors:** Inti Zlobec, Markus Borner, Alessandro Lugli, Daniel Inderbitzin

**Affiliations:** ^1^Institute of Pathology, University of Bern, Murtenstrasse 31, 3010 Bern, Switzerland; ^2^Department of Oncology, Hospital Centre Biel, 2502 Bienne, Switzerland; ^3^Clinical Pathology Division, Institute of Pathology, University of Bern, Murtenstrasse 31, 3010 Bern, Switzerland; ^4^Department of Visceral and Transplantation Surgery, Inselspital-Bern University Hospital, 3010 Bern, Switzerland

## Abstract

The presence of tumor budding (TuB) at the invasive front of rectal cancers is a valuable indicator of tumor aggressiveness. Tumor buds, typically identified as single cells or small tumor cell clusters detached from the main tumor body, are characterized by loss of cell adhesion, increased migratory, and invasion potential and have been referred to as malignant stem cells. The adverse clinical outcome of patients with a high-grade TuB phenotype has consistently been demonstrated. TuB is a category IIB prognostic factor; it has yet to be investigated in the prospective setting. The value of TuB in oncological and pathological practice goes beyond its use as a simple histomorphological marker of tumor aggressiveness. In this paper, we outline three situations in which the assessment of TuB may have direct implications on treatment within the multidisciplinary management of patients with rectal cancer: (a) patients with TNM stage II (i.e., T3/T4, N0) disease potentially benefitting from adjuvant therapy, (b) patients with early submucosally invasive (T1, sm1-sm3) carcinomas at a high risk of nodal positivity and (c) the role of intratumoral budding assessed in preoperative biopsies as a marker for lymph node and distant metastasis thus potentially aiding the identification of patients suitable for neoadjuvant therapy.

## 1. Introduction 

**Figure 1:**
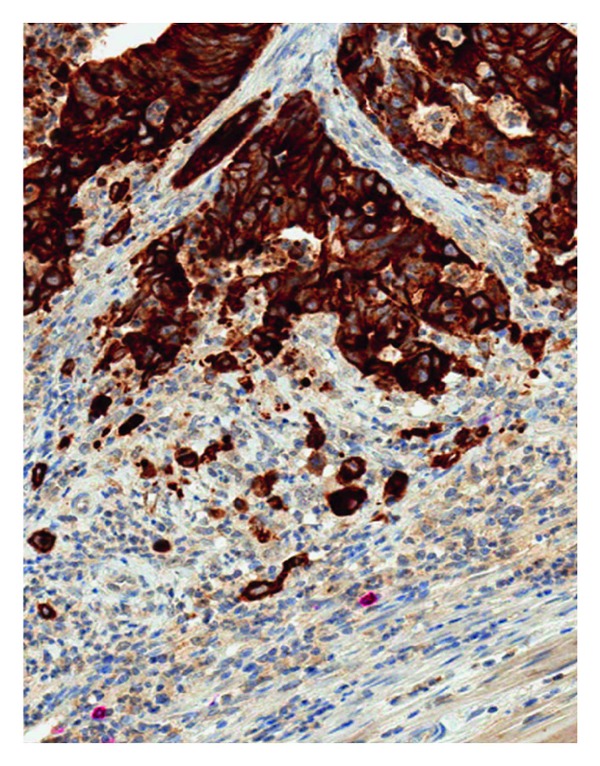
Immunohistochemical analysis highlighting the presence of peritumoral buds at the invasion front of rectal cancer (pancytokeratin stain: CK22, 40x magnification).

Tumor budding (TuB) refers to the presence of detached single tumor cells or clusters of up to 5 cells scattered within the stroma at the invasive tumor front of many different solid cancers [1]. TuB as a histomorphological feature is best described in gastrointestinal tumors and was first comprehensively investigated by Jass in the mid 1980s in patients with rectal cancer [2]. TuB can be evaluated at high magnification using regular H&E staining but its visualization is markedly facilitated with the use of pan-cytokeratin stains (Figure 1).

It is hypothesized that tumor buds, or at least a subpopulation of these cells, have undergone a process similar to epithelial mesenchymal transition (EMT) and have acquired the ability to act as malignant stem cells [3]. Immunohistochemical staining of tumor buds in colorectal cancers shows a clear overexpression of markers involved in extracellular matrix degradation, angiogenesis, migration, and invasion and decreased Ki67 staining indicative of a low proliferation rate [4]. An overexpression of nuclear beta-catenin and simultaneous loss of cell adhesion markers, in particular, E-cadherin is classically observed in tumor-budding cells [5]. 

With such an aggressive phenotypic constellation, it is not surprising that TuB has consistently been linked to lymph node positivity, the presence of lymphatic and venous invasion, as well as with the presence of distant metastatic disease [6–12]. The frequency of high-grade TuB in colon and rectal cancer varies; it has generally been reported to occur in 25–60% of all cases but is correlated with disease progression [7, 9, 13–15]. For example, high-grade TuB is reported in 15–17% of patients with early pT1 tumors [8, 15, 16], 26% of pT2 cases [17], 36–51% of pT3 tumors [12, 17, 18], and up to 73% of pT4 cancers [18]. In addition, it occurs significantly more frequently in patients with node-positive tumors (51–75%) in comparison to patients with TNM (6th ed.) stage II (T3/T4, N0) (15–29%) cancers [8, 15, 17]. Currently, TuB is listed as a category IIB prognostic factor and many studies have confirmed that the presence of TuB is associated with poorer overall and disease-specific and disease-free survival, in most cases independently of the TNM stage [19]. 

The value of TuB in oncological and pathological practice goes beyond its use as a simple histomorphological marker of tumor aggressiveness. In this paper, we outline three situations in which the assessment of TuB may have direct implications on treatment within the multidisciplinary management of patients with rectal cancer. These include (a) the identification of patients with TNM stage II disease potentially benefitting from adjuvant therapy, (b) the identification of patients with early submucosally invasive (T1) carcinomas at a high risk of nodal positivity, and (c) the use of tumor budding as a marker of prognosis and predictor of local and distant relapse assessable in preoperative biopsies.

### 1.1. Stage II Rectal Cancer Patients

Stage II colorectal cancer patients represent a clinically heterogeneous group. Data from the SEER (1975–2005) Public Use File show 5-year survival trends for patients with colon and rectal cancer [35]. In particular for the latter, 5-year overall survival rates decrease dramatically from 64.5% for IIA (T3N0), to 51.6% for IIB (T4aN0) and 32.3% for IIC (T4bN0). Generally, patients with stage II colorectal cancer are not typically considered for additional adjuvant therapy without the presence of additional high-risk features such as perforation or venous and lymphatic invasion [36]. It is suggested, however, that a subgroup of patients with stage II disease who would otherwise have unfavorable clinical outcome and high-risk for metastasis may in fact benefit from adjuvant therapy but the identification of such patients using histomorphological or molecular markers is unclear [37]. 

Although the prognostic effect of high-grade TuB has been well described, studies focusing on the subgroup of stage II cancers are few. Kevans and colleagues evaluated 258 patients with stage II disease and the correlation of TuB with survival and with expression of EMT-related protein markers [38]. They showed that TuB was the only independent marker of poor outcome and had a major effect on the relative risk (RR) of death; patients with high-grade TuB were nearly 8 times more likely to die of disease compared to patients with low-grade TuB. Wang et al. performed a study using 128 patients and evaluated 5-year cancer-specific survival [12]. They show a significant reduction in survival from 91% to 63% in patients with low- versus high-grade TuB and a RR of death of 4.76. Nakamura and colleagues studied 5- and 10-year survival rates for 200 stage II patients as well as the association of TuB on the presence of distant metastasis [9]. Again a substantial reduction in 5-year (93.9% and 73.9%) and 10-year (90.6% and 67.8%) survival time was observed in patients with low-grade versus high-grade TuB tumors. Tanaka and colleagues confirm this finding, reporting disease-specific survival rates of 98% versus 74% in patients with and without TuB, respectively, [10]. Moreover, TuB in stage II patients has been shown to be independent of other prognostic features [13, 39]. An increased frequency of liver and peritoneal metastasis was noted in the high-grade TuB group [9]. Earlier studies show that the sensitivity and specificity of high-grade TuB for distant metastatic disease in patients with stage II tumors are 0.76 and 0.739, respectively, [14]. Frequencies of local recurrence are significantly higher in patients with high- versus low-grade TuB (48% versus 4.5%, resp.) [10]. Finally, the presence of TuB has been significantly associated with isolated tumor cells in lymph nodes of patients with stage II disease in both univariate and multivariate analysis [40].

Taken together, these results strongly suggest that TuB in patients with stage II colorectal cancers has the potential to contribute independent prognostic information. It is linked to more aggressive tumor behavior and is associated with local and distant metastasis. These findings indicate that TuB should be considered as an important histomorphological parameter and may be worthy of investigation and inclusion in prospective clinical trials of patients with stage II disease.

### 1.2. Tumor Budding in Early Rectal Cancers

An important issue in the management of patients with submucosally invasive (T1) colorectal carcinomas is the identification of patients after endoscopic resection that may be at “high risk” for lymph node positivity and thus likely to benefit from surgical resection. The rate of lymph node positivity in this setting is low, approximately 10–15% [23, 24, 27, 29]. Nonetheless histomorphological features capable of predicting lymph node involvement are highly sought after. 

TuB has been shown in several studies to have predictive power for lymph node involvement in either univariate or multivariate analyses (Table 1). In addition to other features such as histological type, lymphatic and venous invasion, TuB is significantly more frequent in cases with lymph node positivity [22, 24–26, 28, 29]. One study evaluated the impact of TuB in T1 cancers and the potential for the development of distant metastasis. In one subgroup of T1 patients eventually developing metastatic disease and a control group of T1 patients with favorable long-term outcome, TuB was significantly more frequent in the metastatic cohort [21]. A different study on 145 patients with T1 cancers used immunohistochemistry and special stains to identify venous invasion, lymphatic invasion, and the presence of TuB by Elastica van Gieson, D2-40 and CAM5 staining, respectively, [25]. In multivariate analysis of lymph node positivity, only venous invasion and TuB were independently predictive of involvement. TuB could predict the presence of distant metastases but only in univariate analysis. 

The examples listed in Table 1 underline the potential importance of the additional assessment of TuB in the pathological diagnosis of early pT1 cancers. TuB assessed in these submucosally invasive carcinomas during daily routine may have a promising role as a histomorphological marker for the prediction of lymph node positivity in this setting.

### 1.3. Is There a Role for Tumor Budding in the Preoperative Setting?

**Figure 2:**
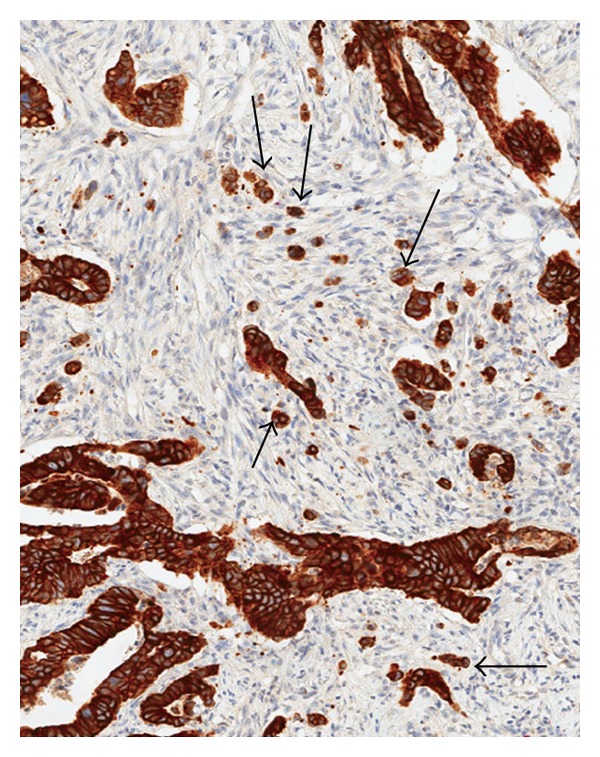
Presence of intratumoral buds (arrows) in the main tumor body of a rectal cancer (pan-cytokeratin stain: CK22, 40x magnification).

Traditionally, the preoperative rectal biopsy can supply three different types of information. The first is the histopathological diagnosis and confirmation of carcinoma, the second is the histological subtype, and the third is the degree of differentiation (tumor grade). However, recently, studies have not only noted the presence of tumor buds within the preoperative biopsy specimen but have also linked this feature to unfavorable prognostic parameters. We have described the presence of tumor budding within the biopsy specimen as “intratumoral” budding (ITB) in order to distinguish it from the classical “peritumoral” budding (PTB) that is located at the invasive front and thus not normally evaluable in biopsy specimens [41] (Figure 2).

The first assessment of tumor budding in rectal cancer biopsies dates to 1989 [42]. Morodomi and colleagues observed that nearly half of all rectal cancer biopsies contained ITB and its presence was a strong indicator of lymph node positivity. Specifically, lymph node involvement was observed in 78.8% of ITB-positive cases and in only 28.1% of ITB-negative rectal cancers. Despite these promising results, the issue of budding within biopsy specimens was only addressed once again in 2011. Using two cohorts of colorectal cancer patients from all stages totaling more than 500 cases, we could confirm the value of ITB as a predictor of lymph node positivity with similar sensitivity (72.7%) and specificity (72.1%) [41]. The presence of ITB not only correlated with vascular invasion but also showed an independent and unfavorable prognostic effect in multivariable analysis. A recent study by Giger et al. evaluated ITB in preoperative biopsies and the predictive values for both lymph node and distant metastasis in a series of 72 colorectal cancers of all TNM stages [43]. Seventeen percent of all cases were found to have high-grade ITB. Of the ITB positive cases, 83.3% had lymph node metastasis and 82% had distant metastasis. This is in contrast to only 51% and 35% of ITB-negative cases, respectively.

A strong linear correlation between the presence of ITB in biopsies and corresponding PTB in resection specimens has been made [41, 43]. This is relevant since the identification of “invasion front” can in some postoperative specimens be challenging. A recent meta-analysis of 42 different histomorphological and immunohistochemical markers in colon and rectal cancers aimed to identify predictors of lymph node metastasis. Focusing on the subset of rectal cancers, Glasgow and colleagues found only two predictive factors, one of which was tumor budding at the invasion front. Again, the sensitivity and specificity of tumor budding for node-positivity across 7 studies with more than 1500 patients were 70% and 69.4% [44]. 

Taken together, the current body of evidence indicates that regardless of its localization, that is, within the main tumor body or invasion front, tumor budding may be a reliable histomorphological predictor of lymph node metastasis and a factor of poor prognosis which can be applied to both postoperative specimen and, most importantly, preoperative biopsy. 

## 2. Conclusion

At least two avenues of investigation should still be clarified before implementing TuB as a criterion for patient management. First, no prospective studies have been conducted to definitely validate the potential of TuB in the clinical decision-making process. Secondly, TuB remains severely underreported in daily diagnostic routine due largely to the absence of a standardized or internationally accepted method for its assessment. Nonetheless, efforts are currently on-going to compare and validate the prognostic effects of TuB using various methods of assessment and in particular their inter- and intraobserver agreement. The evidence supporting an important role of TuB in the clinical and multidisciplinary management of patients with rectal cancer, for example in the setting of stage II and submucosally invasive tumors continues to grow. Although less than a handful of studies have evaluated the presence of intratumoral budding from the preoperative rectal biopsy, the ability to predict, with high accuracy, the presence of lymph node metastases in the pretreatment setting would be of considerable clinical value.

## Figures and Tables

**Table 1:** Summary of studies evaluating tumor budding in submucosally invasive (T1) colorectal carcinomas.

Ref.	Number of patients	Endpoint	Summary of relevant findings
[20]	499	LN+	8.2% of T1 were LN+. Several features were independent predictors of LN+: tumor differentiation/mucinous histology, depth of submucosal invasion, venous invasion, and TuB.
[6]	111	LN+	TuB was associated with LN+ in univariate but not multivariate analysis when analysed with protein markers.
[21]	32	DM	In comparison to a control group, TuB was more frequent in patients who eventually had a distant metastasis in univariate but not multivariate analysis.
[22]	111	LN+	Several features were evaluated including lymphatic and venous invasion, submucosal depth, histologic type, and TuB. In multivariate analysis, only histologic type and TuB predicted LN+.
[23]	65	LN+	T1-T2 rectal cancers. 6.9% of T1 were LN+. TuB predicted lateral LN+.
[24]	322	LN+	14.3% of T1 were LN+. Several features predicted LN+: invasion depth, lymphatic and venous invasion, tumor differentiation, growth pattern, and TuB. Only lymphatic invasion, differentiation, and TuB were independent predictors in multivariate analysis.
[25]	124	LN+ and DM	Elastica von Gieson, D2-40, and CAM5 were used to enhance visualization of venous invasion, lymphatic invasion, and TuB, respectively. TuB was an independent predictor of LN+ and DM+ in multivariate analysis.
[26]	87	LN+ and LR	Prospective study evaluating two groups of patients after endoscopic resection: a surgical group and a follow-up group without surgery. TuB was the only independent predictor of LN+ in multivariate analysis.
[27]	164	LN+	9.8% of T1 were LN+. TuB was significantly associated with LN+ in univariate and multivariate analysis.
[28]	71	LN+	Tumor size, depth of invasion, histologic type, TuB, and lymphatic invasion were predictors in univariate analysis but only TuB and lymphatic invasion were significant in multivariate analysis.
[29]	86	LN+	13% of T1 were LN+. Vascular invasion, tumor budding, and degree of submucosal invasion could be combined to strongly predict LN+.
[30]	214	LN+	Several histopathological and protein markers were evaluated. TuB was a significant predictor in univariate and multivariate analysis.
[31]	76	LN+	TuB can be used in a predictive equation for LN+.
[32]	56	LN+	TuB evaluated using CAM5 was significantly more frequent in LN+ (16/42) than LN negative (0/14) cases.
[16]	159	LN+, OS	10.1% of T1 were LN+ and were associated with several features including TuB. TuB did not influence overall survival.
[33]	51	LN+, LR	TuB was associated to lymphatic invasion, LN+, and local relapse.
[34]	79	LN+	13.9% were LN+. TuB was one of five risk factors for LN+.

TuB: tumor budding; LN: lymph node; DM: distant metastasis; LR: local recurrence; OS: overall survival.
